# Oral management of a patient with down syndrome and agammaglobulinemia: a case report

**DOI:** 10.1186/s12903-020-1056-2

**Published:** 2020-03-13

**Authors:** Yasuka Kusumoto, Kohsuke Imai, Yoshio Ohyama, Haruhisa Fukayama, Osamu Shinozuka

**Affiliations:** 1 Department of Dentistry for Persons with Disabilities, Graduate School of Medical and Dental Sciences, Tokyo Medical and Dental University, 1-5-45, Yushima, Bunkyo-ku, Tokyo, Japan; 2 Department of Community Pediatrics, Perinatal and Maternal Medicine, Graduate School of Medical and Dental Sciences, Tokyo Medical and Dental University, Tokyo, Japan; 3 Department of Oral and Maxillofacial Surgery, Shizuoka City Shizuoka Hospital, Shizuoka, Japan; 4 Department of Anesthesiology and Clinical Physiology, Graduate School of Tokyo Medical and Dental University, Tokyo, Japan

**Keywords:** Down syndrome, Immunodeficiency, Oral management, Case report

## Abstract

**Background:**

Down syndrome is characterized by a variety of dysmorphic features and congenital malformations, such as congenital heart disease, gastrointestinal disease, and other conditions like leukemia and autoimmune disorders. Patients with Down syndrome are highly prone to respiratory tract infections, which might be fatal to them. However, there are only few available data on patients diagnosed with Down syndrome and agammaglobulinemia. In this report, we describe a case of successful prevention of post-dental treatment complications (e.g., pneumonia and other bacterial infections) in a patient with Down syndrome and agammaglobulinemia.

**Case presentation:**

A 43-year-old man with Down syndrome, untreated agammaglobulinemia, and a history of recurrent pneumonia, was referred to our clinic for tooth mobility. To reduce the risk of post-operative infections, gammaglobulin treatment and prophylactic administration of antibiotics was scheduled before the dental procedure. Furthermore, the dental treatment, which included a filling and extractions, was conducted under general anesthesia and with the supervision of a hematologist. The dental procedures were successfully performed without any post-operative infection, and the patient is undergoing follow-up care.

**Conclusions:**

The purpose of this case report was to recommend a close liaison between physicians and dentists who may encounter a similar case, and to emphasize the importance of improving oral health of immunodeficient patients to prevent infections caused by oral microbial flora.

## Background

Down syndrome is characterized by a variety of dysmorphic features and congenital malformations, such as congenital heart disease, gastrointestinal disease, and other conditions like leukemia and autoimmune disorders. Patients with Down syndrome are highly prone to respiratory tract infections, which constitute the most important cause of mortality in such patients at all ages [1, 2]. Agammaglobulinemia is one of the most common primary immunodeficiency disorders, and is characterized by the absence of immunoglobulins. In 1952, Bruton was the first physician to describe a clinical case of absence of immunoglobulins [3]. The diseases of primary antibody deficiency (PAD) represent a class of disorders in humans in which a defective immune system fails to produce antibodies. Patients with PAD generally have low levels of immunoglobulin (Ig) A, IgG, or IgM. PAD is associated with frequent infections of the respiratory tract, skin, sinuses, and lungs. Therefore, there is a high risk of infection during dental treatment in a patient with PAD. However, there is no report of dental treatment in patients with Down syndrome diagnosed with agammaglobulinemia.

Here, we report the successful prevention of post-dental treatment complications, such as pneumonia and other bacterial infections, in a 43-year-old man with Down syndrome and agammaglobulinemia, through immunoglobulin administrations and prophylactic antibiotherapy.

## Case presentation

Consent for publication in this report was obtained from the patient’s mother.

**Fig. 1:**
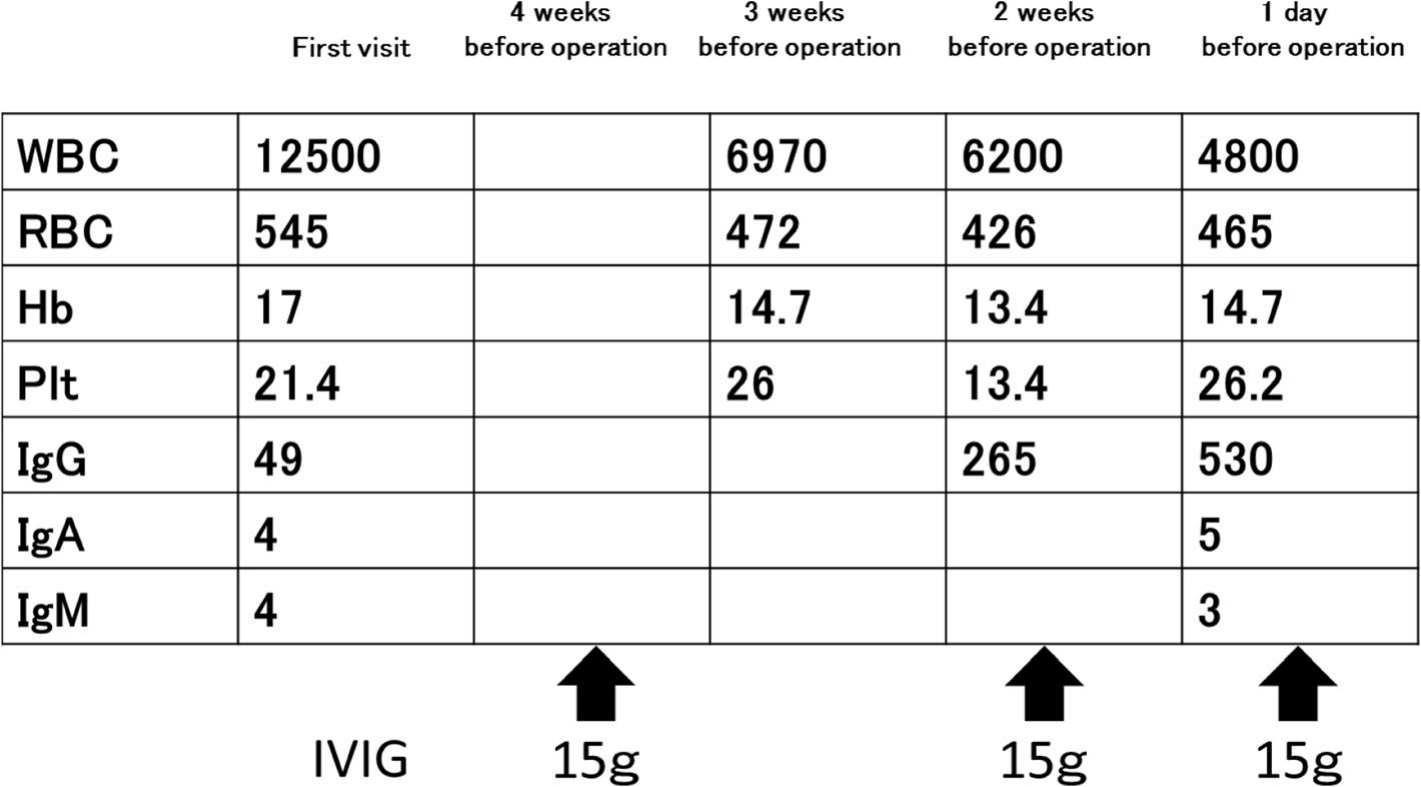
Schedule of pre-operative intravenous immunoglobulin (IVIG) therapy. Hb = hemoglobin level (g/dL); IgA = immunoglobulin A (mg/dL); IgG = immunoglobulin G (mg/dL); IgM = immunoglobulin M (mg/dL); IVIG = intravenous immunoglobulin substitution; Plt = platelet count (10,000/μL); RBC = red blood cell count (10,000/μL); WBC = white blood cell count (/μL)

**Fig. 2:**
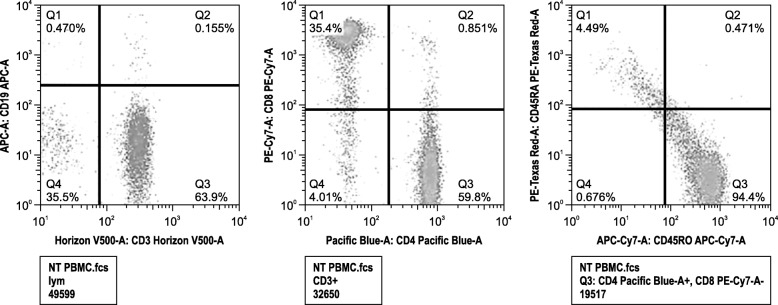
Flow cytometric analysis of the patient’s peripheral blood mononuclear cells (PBMCs). PBMCs from the patient were stained with monoclonal antibodies for CD19, Cd3, CD4, CD45RA, and CD45RO. The B-cells and naive T-cells were remarkably decreased in the patient

A 43-year old male patient was referred to the Clinic for Persons with Disabilities at the Dental Hospital of Tokyo Medical and Dental University (Tokyo, Japan) with a primary complaint of tooth mobility. He had a history of Down syndrome that was diagnosed at birth. He lived alone with his mother. Family history was unremarkable. He had experienced recurrent pneumonia and chronic bronchitis since he was 34 years old. Subsequent immunological assessment revealed agammaglobulinemia (Fig. 1) and B-cell deficiency (0.47%) associated with decreased CD45 RA+ naive CD4+ T-cells (4.5% of CD4+ T-cells) (Fig. 2). He had never received gammaglobulin treatment.

**Fig. 3:**
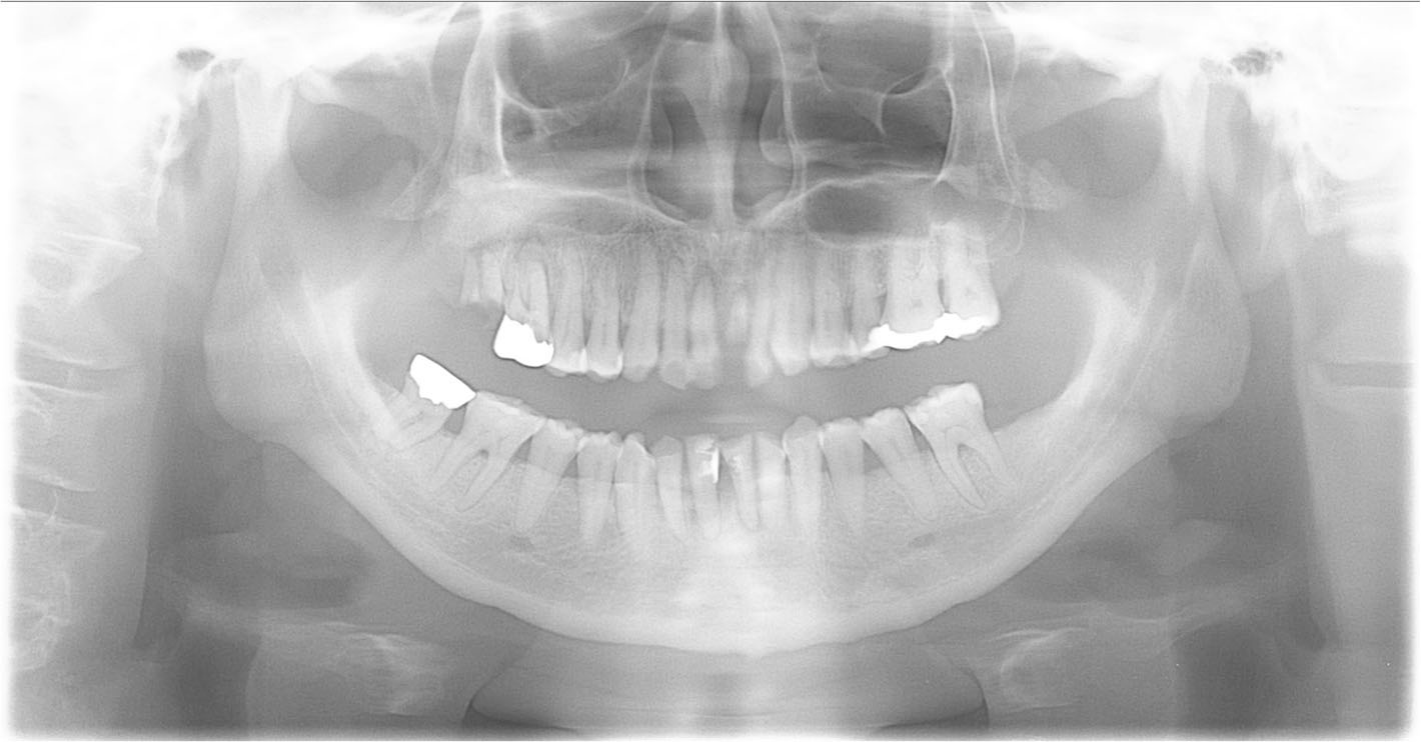
Panoramic radiograph, radiographic examinations revealed alveolar bone resorption in maxillary incisors and several decayed teeth

Oral and radiographic examinations revealed alveolar bone resorption in maxillary incisors and several decayed teeth (Fig. 3). Marginal gingivitis was observed all around the teeth. The patient’s oral hygiene was very poor with dental plaque on all surfaces of his teeth.

The patient had severe mental retardation and autistic features, which included difficulty in communication. Thus, with the consent of his family, it was decided to perform a comprehensive evaluation and treatment under general anesthesia as an in-patient procedure. After consultation with a hematologist, the patient received three courses of intravenous immunoglobulin (IVIG) therapy to restore and maintain his serum IgG levels above 500 mg/dL (Fig. 1). IVIG therapy was implemented at 4 weeks, 2 weeks, and 1 day before operation.

The mandibular right first molar was restored with light-cured composite resin. The maxillary left first molar, second molar, maxillary incisors, and mandibular left incisor were extracted. After extraction, sockets were sutured to prevent post-operative infection. Suture reduced the risk of rebleeding and relieved patient discomfort.

Operating table was prepared in the usual fashion. For operative field, we used 0.025% benzalkonium chloride solution and normal saline solution as usual. All procedures were carried out under standard disinfection without any additional measures.

Ampicillin sodium (6 g/day) was administered every 12 h intravenously, beginning in the morning before the operation and then for 4 days after the operation, following which the patient was discharged without any infection or complication.

At present, the patient is undergoing follow-up care, and the marginal gingivitis has improved. He is receiving regular IVIG treatments under the care of his local physician.

## Discussion

The case reported here was successfully managed through the administration of gammaglobulin and antibiotics. This report describes the management of agammaglobulinemia in a patient with Down syndrome during oral care procedures. Autoimmune diseases are frequently observed in patients with Down syndrome, with prevalence of immune deficiency, mild to moderate T-cell and B-cell lymphopenia with decreased naive lymphocytes, impaired mitogen-induced T-cell proliferation, reduced specific antibody responses to immunizations, and defects in neutrophil chemotaxis [4, 5]. These abnormalities may contribute to increased susceptibility to viral infections, hematologic malignancies, and autoimmune diseases associated with Down syndrome [4, 5]. For invasive dental procedures, such patients are at a high risk of severe infection and septicemia caused by the spread of oral microorganisms and their toxins through circulating blood. Clinical use of IVIG therapy has increased in the treatment of patients with PADS. IVIG therapy in patients with agammaglobulinemia reduces the risk of infection [6, 7]. It involves therapeutic preparations of pooled polyspecific IgG, obtained from the plasma of a large number of healthy individuals. IVIG approach had a significant and positive therapeutic impact in our patient (Fig. 1).

IVIG therapy prevents many, though not all, pulmonary complications. Though they are receiving IVIG therapy, in some patients with relatively more severe antibody deficiencies, may be in high risk of chronic bacterial infections [8]. Then a standard course of antibiotics for acute infections stemming from surgical treatment would not be sufficient in severely immunodeficient patients and may lead to rapid relapse or recurrence of infections and further morbidity, including permanent scarring and loss of function. Experienced clinical immunologists often prescribe a course of antimicrobials that are two to three times longer than standard recommendations [9, 10]. In the case described here, antibiotic prophylaxis besides the induction of IVIG treatment was effective for dental treatment of the immunodeficient patient.

Owing to their susceptibility to infection, immunodeficient patients require precautions during dental treatment. Dental treatment of patients with severe combined immunodeficiency has not been previously reported in dental or medical literature. Based on our experience with this patient, we recommend that IgG, IgA, and IgM should be evaluated in patients with Down syndrome before they undergo dental procedures. Delayed diagnosis of agammaglobulinemia and other PADs might result in frequent hospitalizations owing to bacterial infections, including pneumonia, which could lead to chronic lung diseases.

In the current case, initial oral examination revealed poor oral hygiene. Home care is essential for a patient’s oral hygiene and dental health. However, this is difficult to achieve in patients with Down syndrome owing to the intellectual impairment and decreased manual dexterity [11]. Since patients with Down syndrome frequently experience respiratory infections, regular oral and dental examination should be performed to reduce the risk of aspiration pneumonia; it should be remembered that as they age, people with Down syndrome are more likely to present with immune deficiency syndromes related to early senescence [12] and innate abnormalities in the immune response. Physicians and dentists should take exceptional precautions to detect oral pathogens in these patients, since the pathogens may result in pneumonia and other severe infection. Early diagnosis and treatment, including IVIG, is essential to improve prognosis and quality of life of patients with PADs [13]. In addition, if possible, newborn mass screening for PADs is recommended [14–17].

## Conclusions

The purpose of this case report was to recommend a close liaison between physicians and dentists who may encounter a similar case, and to emphasize the importance of periodontal health in immunodeficient patients to prevent infections caused by oral microbial flora.

## Data Availability

The datasets generated and analyzed during the current study are not publicly available since they contain medical information of the patient.

## Abbreviations

IgA: Immunoglobulin A
IgG: Immunoglobulin G
IgM: Immunoglobulin M
IVIG: Intravenous immunoglobulin
PAD: Primary antibody deficiency
